# A retrospective database study of the demographic features and glycemic control of patients with type 2 diabetes in Kinshasa, Democratic Republic of the Congo

**DOI:** 10.1186/s12916-022-02458-2

**Published:** 2022-08-19

**Authors:** Diana Sagastume, Elly Mertens, Deogratias Katsuva Sibongwere, Jean-Claude Dimbelolo, Jean Clovis Kalobu Kabundi, Jeroen de Man, Josefien Van Olmen, José L. Peñalvo

**Affiliations:** 1 Unit of Non-Communicable Diseases, Department of Public Health, Institute of Tropical Medicine, Nationalestraat 155, 2000 Antwerp, Belgium; 2 Centre d’Éducation Diabète & Santé Boulevard Lumumba n° 1 Musoso district, Municipality of Limete, Kinshasa, Democratic Republic of the Congo; 3 Memisa, 19 Square de Meeûs, 1050 Brussels, Belgium and Memisa representation in Kinshasa, 47 Kisangani, commune de la Gombe, Kinshasa, Democratic Republic of the Congo; 4 Department of Family Medicine and Population Health, University of Antwerp, Doornstraat 331, 2610 Wilrijk, Belgium

**Keywords:** Type 2 diabetes, Glycemic target, Metabolic risk factors, Longitudinal analysis, Primary care database

## Abstract

**Background:**

The Kin-Antwerp project aimed at improving the quality of care provided to patients with diabetes in Kinshasa, the Democratic Republic of the Congo in Central Africa, including the digitalisation of routine clinical data to improve patients’ follow-up. We aim to analyse the data of a study population of Kin-Antwerp to characterise their demographic features, assess their achievement of glycemic target over time, and identify groups requiring prioritised attention.

**Methods:**

We performed a secondary database analysis of routinely collected information from primary care patients with type 2 diabetes followed from 1991 to 2019. Data included demographics (age, sex), anthropometrics (weight, height), clinical parameters (blood pressure, plasma glucose), and anti-diabetic treatments. Achievement of glycemic target, defined as fasting plasma glucose < 126 mg/dL, over time was assessed using a multilevel mixed-effects logistic regression model.

**Results:**

Our study population of patients with type 2 diabetes (*N* = 8976) comprised a higher proportion of women (67%) and patients between 40 and 65 years old (70.4%). At the first follow-up, most patients were on treatment with insulin (56.5%) and had glycemic levels under the target (79.9%). Women presented more often with obesity (27.4%) and high systolic blood pressure (41.8%) than men (8.6% and 37.0%, respectively). Patients had a median follow-up of 1.8 (interquartile range: 0.5–3.4) years. Overall, the odds of achieving glycemic target increased by 18.4% (odds ratio: 1.184, 95% CI: 1.130 to 1.239; *p* < 0.001) per year of follow-up. Stratified analyses suggested that the odds of achieving glycemic control over time increased among older patients (> 40 years), but not among younger patients (< 40 years).

**Conclusion:**

In our study population, an overall poor glycemic control was observed albeit with a modest improvement in the achievement of glycemic target throughout patients’ follow-up. Younger patients may benefit from prioritised attention to achieve glycemic targets. Based on the information provided by the database, continue monitoring and improvement of the project Kin-Antwerp is recommended. Introducing further efforts to improve type 2 diabetes management should include robust glycemia-monitoring tools and haemoglobin A1c, as well as further outlining self-management strategies.

**Supplementary Information:**

The online version contains supplementary material available at 10.1186/s12916-022-02458-2.

## Background

Diabetes is a leading cause of morbidity and mortality and one of the major constraints to health systems worldwide [1, 2]. In 2019, the International Diabetes Federation (IDF) reported a global prevalence of diabetes of 9.3% for the adult population (18–79 years) [3]. This reported prevalence is greater in high-income (10.4%) than middle- (9.5%) and low- (4.0%) income countries, however, these numbers are expected to rise rapidly among those in the latter regions where economies are generally moving towards improvement [3]. In Africa, the prevalence of type 2 diabetes (T2D) has been increasing rapidly and is predicted to continue, likely driven by the increases in body weight and adiposity [4] as a consequence of unhealthy lifestyles, rapid urbanisation, and overall population ageing [5]. Estimations from 1980 to 2014 for the entire African continent have reported an increase in the prevalence of T2D of more than double for both women (4.1 to 8.9%) and men (3.4 to 8.5%) [4] and such increases being associated with the growing rates of obesity [6]. These estimates are, however, based on limited population-based data, as health data remain scarce in several low- and middle-income countries (LMICs) in Africa, particularly in those in sub-Saharan Africa (SSA) [7, 8]. This shortcoming has likely resulted in an underestimating of the burden of T2D across this region. In the Democratic Republic of the Congo (DRC), the IDF has estimated an age-adjusted prevalence of diabetes of 6% for adults (20-79 years) [3]. However, studies conducted in specific populations, such as mining workers in DRC, has reported a much higher prevalence of 11.9% [9].

Previous evidence from different African countries has reported an overall poor glycemic control (haemoglobin A1c (HbA1c) ≥ 7% and fasting plasma glucose (FPG) ≥ 126 mg/dL) in patients with T2D [10–12]. Moreover, it has been described that the sub-optimal management of T2D in LMICs is often related to the limited accessibility and availability of anti-diabetic drugs, and fragmented delivery of chronic care [13].

To address these concerns, availability and access to health data in DRC and improve the quality of care, international projects like Kin-Antwerp, a collaboration between the Institute of Tropical Medicine Antwerp (ITM), Memisa (Belgian NGO with representation in DRC), and Centre d’éducation diabète & Santé in Kinshasa, started in 2010. This project aimed at supporting the ongoing DRC Diabetes National Programme to improve health services and provide a closer follow-up to patients with T2D by centralising data collection. The latter focused on the digitalisation of routine clinical data collected during follow-up visits through the development of a software database. Thus, the primary aims of this study are to describe the demographic characteristics of a study population of patients with T2D of Kin-Antwerp, assess their overall achievement of glycemic target over the years of follow-up, and identify potential subgroups, based on sex and age, that may require prioritised attention. Through this, we also aim at providing an insight into the effectiveness of the project Kin-Antwerp. This study is an exploratory analysis; therefore, no pre-specified hypotheses were investigated.

## Methods

### Design

This research is a secondary database study of routine data collected from patients with T2D attending primary care and followed from 1991 to 2019. This research was approved by the Institutional Review Board of the Institute of Tropical Medicine Antwerp in Belgium (IRB/RR/ac/149) and the Ethical Committee of the University of Kinshasa in DRC (ESP/CE/153B/2021). This study was guided by the Reporting of studies Conducted using Observational Routinely-collected health Data (RECORD) Statement [14], an extension from STROBE, The Strengthening the Reporting of Observational Studies in Epidemiology, guideline [15].

### Setting

According to information and data from the World Bank in 2020, DRC is the largest country of SSA with approximately 89,561,404 million inhabitants and has a gross domestic product (GDP) annual growth of 1.7% [16, 17]. In 2018, it was estimated that DRC has one of the largest populations living in poverty, precisely the third one globally, as approximately 73% of the population lives with less than $1.90 a day [18].

In 2015, the World Health Organization (WHO) has described that DRC has a 3-level health system organisation: the implementation level, each district in DRC has a network of several health centres and district hospitals; the intermediate level focuses on the technical and logistic support and is mainly managed by provincial health departments; the central level has the normative responsibility [19]. Between 2008 and 2012, the health expenditures in DRC were rather low, $12–13 per capita per year [19]. A large part of the challenges of the health system in DRC originates in the political situation of three decades of non-governance resulting in the collapse of the state and economy [19].

In the big capital city of DRC, Kinshasa, adults seek medical help close to their homes when health complaints are present. During medical check-ups at primary health facilities, screening for T2D is typically done through medical history, clinical parameters (e.g. blood pressure and blood glucose) and anthropometric measurements such as body weight and height. When blood glucose is abnormal (random glucose test of > 200 mg/dL or FPG > 126 mg/dL) and/or diabetes-related symptoms are present, for instance, polyuria, polyphagia, or polydipsia, extreme fatigue or blurred vision, patients are referred, in most of the cases, to the endocrinology department of the hospital for further testing. After the diagnosis of T2D is confirmed and the physician has selected the most appropriate treatment, the national standard form for patients with T2D is completed. Developed by the Diabetes National Programme, this paper-based form collects the diabetes history of the patient, demographic details, diagnosis status, clinical evaluation, and anti-diabetic treatment records. Diagnosis of T2D is carried out by a doctor at the hospital or by a nurse at the primary health centres. If the diagnosis was performed at the hospital, from the moment the patient’s glycemia is stable, the continuation of treatment and care is decentralised to the primary health centres.

In Kinshasa, T2D care is often offered by Kin Réseau, a network initiative, which was set up in 1974 by mostly religious organisations, aiming at providing decentralised care for diabetes. This longstanding network, comprises 80 care centres, including hospitals and primary health centres, and has a patient referral system in place. This programme also offers patient education, medication for a subsidised price, and daily insulin administration at the health centre, as well as annual screening for complications of diabetes. More details on this programme can be found elsewhere [20]. Currently, Kin Réseau offers a basic diabetes package that includes one follow-up visit a week at the health centre and a bi-monthly medical examination by a trained doctor for a price of USD 3.5/month.

### Data sources

The Kin Réseau network often provides T2D care by offering medical packages. The T2D care package includes disease follow-up visits in which routine measurements such as weight, blood pressure, and foot examinations are assessed*.* Glycemic control is monitored by measuring FPG. Based on clinical assessment and test results, the physician decides on treatment adjustments. Patients are encouraged to achieve glycemic targets recommended by the IDF of HbA1c < 7.0 % and its equivalent of FPG < 126 mg/dL [21]. The routine data gathered through these visits is collected in a paper-based follow-up form used systematically across DRC (additional file, Image S1). These forms have been collected and stored by the Centre d’éducation diabète & santé covering the years between 1991 and 2019. In the context of Kin-Antwerp’s main objective, supporting Kin Réseau in improving the quality of care and a better follow-up of patients, a database software was developed by the ITM in collaboration with staff from Memisa and the Centre d’éducation diabète & santé. After its development, staff from both Congolese institutions were trained on the use of the database and data entry and, throughout the years, the database has evolved based on data quality controls. Currently, the established electronic database is an independent effort from the Centre d’éducation diabète & santé and is limited to the information collected in the follow-up form (Image S1) of patients with T2D. Paper-based forms with information collected at 6-month follow-up visits, as this cut-off was set to indicate if a patient has not attended for disease follow-up, of patients with T2D have been entered retrospectively into the database from the latest forms received in 2019. Currently, the database contains information on approximately 13,000 patients. Data were checked for missingness and accuracy. Data-cleaning processes were carried out before the analyses and included removing irrelevant data, standardising terms and fixing typing errors, and converting data types. This study did not include any data linkage.

### Study population and variables

Kin-Antwerp gathers information from 65 health centres across Kinshasa, out of which 32 centres had updated information on patients with T2D. For this study, code was developed to retrieve information based on the following eligibility criteria. Data on adults (≥ 18 years old) diagnosed with T2D were included (*N* = 9700; 41,353 observations). Patients’ information was excluded if any of the following variables were not available at the first visit: date of the visit (0.3%), sex (0.0%), glycemic value (1.9%), and treatment (7.8%) leaving information for a total of 8976 patients with multiple follow-up visits representing 37,548 observations. The index date was defined as the date of the first prescription of anti-diabetic medication for a patient meeting the inclusion and exclusion criteria in the database.

Demographic and clinical information described in the database included sex, age, and values at each follow-up visit for weight and height (the latter only at the first visit), and the clinical parameters of systolic and diastolic blood pressure (SBP, DBP; in mmHg) and FPG (mg/dL). Weight was measured in kilogrammes with a digital or mechanical scale placed on a firm and flat surface. Height was assessed using a measuring board positioned against a wall and taken in centimetres. For both measurements, weight and height, standard guidelines developed by each health centre in collaboration with the Centre d’éducation diabète et santé were followed. BMI was calculated as body weight in kilogrammes divided by height in metres squared and classified based on the WHO classification of adults (normal: BMI < 25 kg/m^2^; overweight: BMI ≥ 25 – < 30 kg/m^2^; obesity: BMI ≥ 30 kg/m^2^) [22]. SBP and DBP were measured using either a digital automatic blood pressure monitor or a sphygmomanometer, depending on the resources of the health centre. FPG was measured by pricking the skin with a lancet to obtain a drop of blood which is placed on a disposable test strip, followed by inserting it in the glucometer to estimate the glycemia in blood.

In this study, the operational definition of T2D relies on the physician’s written diagnosis by which patients were referred for follow-up in primary care centres using the standard national forms developed by the National Programme of Diabetes in DRC. Our primary outcome is the odds/probabilities of achieving glycemic target defined as FPG < 126 mg/dL. All available FPG values per patient were used to estimate the achievement of the glycemic target. Secondary outcomes entailed identifying demographic characteristics (sex and age) that could potentially influence the odds/probabilities of achieving glycemic target.

### Statistical analysis

All analyses were conducted using STATA (Release 16/SE. College Station, TX: StataCorp LP). Demographic characteristics were reported as measures of central tendency for continuous data, mean ± standard deviation (SD) and medians and interquartile range (IQR) if not normally distributed, and counts and percentages for categorical variables. To assess the achievement of glycemic target (FPG < 126 mg/dL) over the years of follow-up, a multilevel mixed-effects logistic model (command *melogit*) was conducted. To account for patients nested within health centres, a random intercept was added for the health centres and to consider repeated measurements, we fitted a random intercept at the patient level and a random slope of the variable representing years of follow-up varying by patient. The results of this model are expressed as odds ratios (OR) and 95% confidence intervals (95% CI) and are conditional to the random effects. From this model, we derived probabilities by predicting the average marginal effects (AME) (command *margins*) and illustrated the average marginal effects at specific time points. AME indicates the average change in the probability, in this case, glycemic control, when *x,* years of follow-up, increases by one unit. We adjusted our model at baseline (first visit of follow-up) for sex, age categories (< 40 years, 40-65 years, > 65 years), BMI (normal, overweight, obesity), SBP (normal, elevated), treatment (oral glucose lowering drugs (OGLDs), insulin, insulin + OGLDs, or diet), and interactions between the years of follow-up and the mentioned covariates. These interactions are referred to as time interactions in the manuscript. We assumed the missingness mechanism was ‘missing at random’. Missing values in the covariates of the model were handled by listwise deletion in a long format, while a direct likelihood approach dealt with missing values in the outcome (i.e. the default strategy for regressions in STATA). To identify subgroups of patients that may do worse in terms of achieving the glycemic target, exploratory stratification models for sex and age categories were carried out. A *p*-value < 0.05 was considered statistically significant for the main model and a *p*-value < 0.008 for the stratified exploratory analyses after applying Bonferroni correction for multiple comparisons. As part of our objective was to assess the achievement of glycemic target, measured as odds/probabilities, over the years of follow-up, we chose a multilevel mixed-effects logistic regression, with random intercept and slope as this model allows for patient’s observations to be analysed as a cluster, hence allowing each participant to have its own starting point (intercept) and time of follow-up (slope). We favoured this approach in comparison to a survival analysis which implies selecting an event (achieving glycemic target) at a specific time, for example, time to the first or the last event. A mixed-effects logistic regression accounts for repeated evaluations of glycemic target over the follow-up time, reflecting what has happened in real practice, and taking into account the potential correlation between them, aside from also allowing adjusting the estimate for relevant covariates.

## Results

**Fig. 1:**
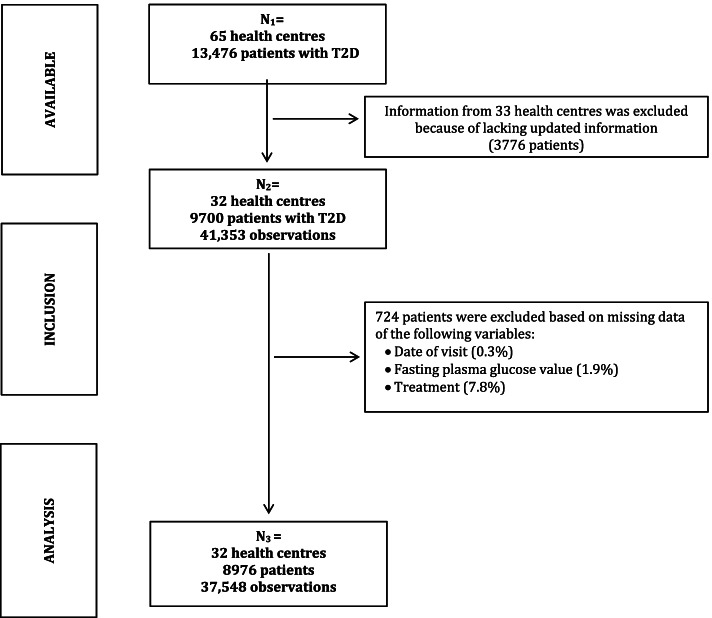
Flow chart—data selection process

The demographics and clinical characteristics of the study population at the first follow-up visit are presented in Table 1. Of a total of 8976 patients (Fig. 1) with T2D, 67% were women and 33% were men with an average age of 55.2 ± 11.3 years old. Most of the patients were diagnosed with T2D 5 to 10 years ago (49.1%), followed by patients diagnosed during the last 5 years (26.7%), fewer were diagnosed between 10 to 20 years ago (22.5%), and hardly any more than 20 years ago (1.7%). Patient follow-up time varied considerably, ranging between 0 and 17.4 years, and the number of follow-up visits ranged from 1 to 27. Patients had a median follow-up years of 1.8 (IQR: 0.5–3.4) and a median number of visits of 3 (IQR: 2–6). The distribution of the number of follow-up visits and years of follow-up is presented in the additional file (Fig. S1 and Fig. S2, respectively).

**Table 1:** Demographic and clinical characteristics of the study population at the first follow-up visit

		Study population (***N*** = 8976; 100%)	Women (***N*** = 6043; 67%)	Men (***N*** = 2933; 33%)
**Demographic characteristics**				
**Age ***mean ± SD*				
^Missing data (1.1%)^	*Years*	55.2 ± 11.3	54.8 ± 11.1	56.1 ± 11.7
**Age classification ***n (%)*				
^Missing data (1.1%)^	*< 40 years*	752 (8.5)	521 (8.7)	231 (8.0)
	*40–65 years*	6248 (70.4)	4294 (71.9)	1954 (67.3)
	*> 65 years*	1873 (21.1)	1157 (19.4)	716 (24.7)
**Diabetes details**				
**Time since diagnosis ***n (%)*				
^Missing data (0.9%)^	*< 5 years*	2379 (26.7)	1595 (26.6)	784 (27.0)
	*5–10 years*	4364 (49.1)	2938 (49.1)	1426 (49.1)
	*11–20 years*	2000 (22.5)	1369 (22.9)	631 (21.7)
	*> 21 years*	149 (1.7)	84 (1.4)	65 (2.2)
Visits*				
*Median [IQR]*	*Number of visits*	3 [2–6]	3 [2–6]	3 [2–5]
Length of follow-up*				
*Median [IQR]*	*Years follow-up*	1.8 [0.5–3.4]	1.9 [0.6–3.4]	1.6 [0.4–3.2]
**Initial treatment ***n (%)*				
^Missing data (0.0%)+^	*OGLDs~*	2949 (32.9)	2066 (34.2)	889 (30.1)
	*Insulin~*	5075 (56.5)	3320 (54.9)	1755 (59.8)
	*Diet*	296 (3.3)	195 (3.2)	101 (3.5)
	*OGLDs + Insulin~*	656 (7.3)	462 (7.7)	194 (6.6)
**Anthropometry**				
**Weight ***mean ± SD*				
^Missing data (2.7%)^	*kg*	69.2 ± 14.5	69.0 ± 14.8	69.4 ± 13.9
**Height ***mean ± SD*				
^Missing data (2.0%)^	*Metres*	1.6 ± 0.1	1.6 ± 0.1	1.7 ± 0.1
**BMI ***mean ± SD*				
^Missing data (4.8%)^	*kg/m*^*2*^	25.9 ± 5.2	26.8 ± 5.5	24.1 ± 4.2
**BMI classification ***n (%)*				
^Missing data (4.8%)^	*Underweight/normal (< 24.9)*	3606 (42.0)	2045 (35.3)	1561 (56.1)
	*Overweight (25–29.9)*	3144 (36.7)	2164 (37.3)	980 (35.3)
	*Obese (≥ 30)*	1827 (21.3)	1587 (27.4)	240 (8.6)
**Clinical parameters**				
**Systolic blood pressure ***mean ± SD*				
^Missing data (1.1%)^	*mmHg*	131.5 ± 24.1	132.7 ± 24.6	129.2 ± 23.0
**Systolic blood pressure ***n (%)*				
^Missing data (1.1%)^	*Normal (< 130 mmHg)*	4022 (45.3)	2610 (43.7)	1412 (48.8)
	*Elevated (≥130 < 140 mmHg)*	1280 (14.4)	869 (14.5)	411 (14.2)
	*High (≥140 mmHg)*	3574 (40.3)	2502 (41.8)	1072 (37.0)
**Diastolic blood pressure ***mean ± SD*				
^Missing data (1.1%)^	*mmHg*	78.9 ± 13.5	79.4 ± 13.7	77.7 ± 12.9
FPG*				
^missing data (0.0%)+ ^*median [IQR]*	*mg/dL*	199 [135–299]	199 [136–297]	199 [133–303]
**Glycemic target ***n (%)*				
^Missing data (0.0%)+^	*Achieved (FPG < 126 mg/dL)*	1807 (20.1)	1201 (19.9)	606 (20.1)
	*Unachieved (FPG ≥ 126 mg/dL)*	7169 (79.9)	4842 (80.1)	2327 (79.3)

*Abbreviations*: *BMI* body mass index, *FPG* fasting plasma glucose, *IQR* interquartile range, *OGLDs* oral glucose lowering drugs

*Values are not normally distributed, therefore, medians and IQR are reported

^+^Missing data equals 0.0% as patients’ information was excluded if any of the following information was not available at the first follow-up visit: date of the visit, sex, glycemic value, and treatment

~The most frequently used type of OGLDs were Metformin and Daonil. For insulin, it varied between mixed, rapid-acting, long-acting, and intermediate-acting insulin

At the first follow-up visit, most patients had initiated treatment with insulin (56.5%), followed by OGLDs (32.9%) or a combination of both insulin + OGLDs (7.3%), while few patients were not prescribed medication and only lifestyle modification was recommended (3.3%). Patients in the database had a mean BMI of 25.9 ± 5.2 kg/m^2^, with 37.3% of women and 35.3% of males classified as overweight and 27.4% and 8.6%, respectively, obese. An average SBP of 131.5 ± 24.1 mmHg and DBP of 78.9 ± 13.5 mmHg was observed, with 14.4% of patients presenting an elevated SBP (≥ 130 to < 140 mm Hg) and 40.3% high SBP (≥ 140 mm Hg). Also, a median FPG of 199 mg/dL (IQR: 135–299) was recorded, with no difference between sex (199 mg/dL (IQR: 136–297) for women and 199 mg/dL (IQR: 133–303) for men), and representing a total of 79.9% of patients above the glycemic target, again, without a clear distinction between women 80.1% and men 79.3%. To provide a broader demographic description of the patients, the demographic characteristics at baseline and the latest follow-up stratified by sex and age groups of a subset of patients with a minimum of 1.5 years of follow-up are presented in the additional file (Table S1 and Table S2, respectively).

### Achievement of glycemic target during the follow-up

**Fig. 2:**
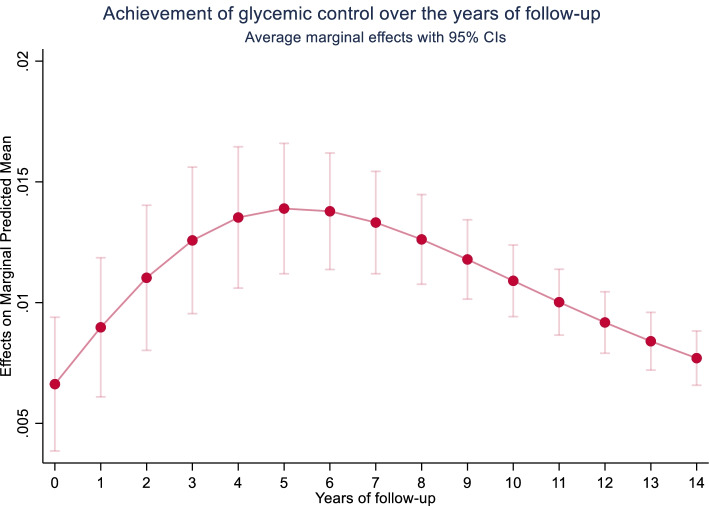
Probabilities and 95% confidence intervals for the achievement of glycemic control at specific time points of the total study population. This graph represents the average marginal effects (the annual marginal predicted means and 95% CI) for achieving the glycemic target at specific time points over the year of follow-up of the total study population. Within a modest overall increase, slightly higher probabilities of achieving glycemic control were observed over the first years of follow-up followed by a decline towards a longer follow-up

Table 2 presents the crude and adjusted multi-level random-effect odds ratios for the achievement of glycemic target over the years of follow-up and the predicted average marginal effects. The adjusted multivariable model including 8648 patients with 35,499 observations (an average of 4.0 time points per patient) showed an increase in the odds of achieving the glycemic target of 18.4% (OR: 1.184, 95% CI: 1.130 to 1.239) per 1-year increase in the follow-up. The average marginal effect per one-year increase was 1.0% (0.010, 95% CI: 0.007, 0.012). In correspondence, Fig. 2 illustrates the average marginal effects with 95% CI of achieving glycemic target at specific time-points over the follow-up. Within the overall modest increase, it depicts an increase in the probability of achieving glycemic control over the first years of follow-up followed by a decline in the probability towards a longer follow-up period. Our model also showed that at baseline, men had a higher odds of achieving the glycemic target (OR: 1.342, 95% CI: 1.218 to 1.478) than women and those in the age groups of 40-65 years had a lower odds (OR: 0.843, 95% CI: 0.755 to 0.942) when compared to individuals > 65 years old. Moreover, patients with overweight appeared to have a higher odds of achieving glycemic target (OR: 1.289, 95% CI: 1.174 to 1.414) as well as those with obesity (OR: 1.306, 95% CI: 1.160 to 1.470) than individuals with a BMI < 25 kg/m^2^. At baseline, compared to insulin-treated patients, those treated with OGLDs (OR: 2.111, 95% CI: 1.913 to 2.331), OGLDs + Insulin (OR: 1.213, 95% CI: 1.015 to 1.449), and diet (OR: 7.717, 95% CI: 6.170 to 9.650) seemed to have a higher odds of achieving glycemic target. Our model revealed significant interactions between the years of follow-up and age groups, years of follow-up and BMI categories, and years of follow-up and treatments.

**Table 2:** Achievement of glycemic target over the years of follow-up

		Achievement of glycemic target—***odds ratio (95% CI)***^1^	Achievement of glycemic target—***probabilities (95% CI)***^2^
**Crude model**	**Years follow-up**	1.041 (1.023, 1.058)	0.009 (0.067, 0.011)
*N = 8976;* *obs = 37,262*		< 0.001*	< 0.001*
**Adjusted model**	**Years follow-up**	1.184 (1.130, 1.239)	0.010 (0.007, 0.012)
*N = 8648;* *obs = 35,499*		< 0.001*	< 0.001
	**Sex**		
	Men	1.342 (1.218, 1.478)	0.054 (0.039, 0.069)
		< 0.001*	< 0.001
	**Age**		
	< 40 years	0.925 (0.766, 1.117)	− 0.050 (− 0.074, − 0.017)
		0.417	0.002
	40–65 years	0.843 (0.755, 0.942)	− 0.047 (− 0.064, − 0.030)
		0.003*	< 0.001
	**BMI**		
	Overweight	1.289 (1.174, 1.414)	0.028 (0.015, 0.040)
		< 0.001*	< 0.001
	Obese	1.306 (1.160, 1.470)	0.025 (0.008, 0.041)
		< 0.001*	0.003
	**SBP**		
	SBP < 130 mmHg	0.946 (0.874, 1.024)	− 0.004 (− 0.015, 0.006)
		0.170	0.423
	**Initial treatment**		
	OGLDs	2.111 (1.913, 2.331)	0.077 (0.062, 0.091)
		< 0.001*	< 0.001
	OGLDs + Insulin	1.213 (1.015, 1.449)	0.022 (− 0.005, 0.048)
		0.034*	0.113
	Diet	7.717 (6.170, 9.650)	0.334 (0.291, 0.377)
		< 0.001*	< 0.001
	**Time-interactions**		
	Sex*years follow-up	1.018 (0.981, 1.057)	–
		0.350	
	< 40 years*years follow-up	0.903 (0.835, 0.976)	–
		0.010*	
	40–65 years*years follow-up	0.943 (0.904, 0.984)	–
		0.007*	
	Overweight*years follow-up	0.959 (0.929, 0.991)	–
		0.011*	
	Obesity*years follow-up	0.944 (0.904, 0.985)	–
		0.008*	
	SBP*years follow-up	1.015 (0.988, 1.043)	–
		0.272	
	OGLDs*year follow-up	0.864 (0.833, 0.897)	–
		< 0.001*	
	OGLDs + Insulin *year follow-up	0.973 (0.901, 1.051)	–
		0.490*	
	Diet* year follow-up	0.909 (0.816, 1.012)	–
		0.082	
	*Variance intercept*^*+*^*(health centre)*	*0.034 (0.016, 0.072)*	–
	*Variance intercept*^*+*^*(ID participant)*	*1.227 (1.112, 1.354)*	*–*
	*Variance slope*^*+*^*(years follow-up)*	*0.041 (0.031, 0.056)*	*–*

*Abbreviations*: *BMI* body mass index, *OGLDs* oral glucose lowering drugs, *SBP* systolic blood pressure

^1^Odds ratio (OR) and 95% CI were obtained from a multi-level mixed-effects logistic regression with two random intercepts (health centres and participants ID) and one random slope (years of follow-up). The odds ratios need to be interpreted conditional on the random effects

^2^Estimated probabilities and 95% CI corresponding to the predicted average marginal effects from the multi-level mixed-effects logistic regression.

The model is adjusted only for baseline (first visit of follow-up) covariates (sex, age, BMI, SBP, initial treatment and their corresponding interactions with years of follow-up). Achievement of glycemia target (achieved < 126 mg/dL and unachieved ≥ 126 mg/dL (reference)

^+^Reported as estimates and corresponding 95% CI. Reference categories for the covariates are the following: sex (women), age (> 65 years old), BMI (BMI < 25 kg/m^2^), SBP (elevated, > 130 mmHg), initial treatment (Insulin), and same reference group was used for the interaction of covariates with years of follow-up. Extra information: average time points per patients for the crude model: 4.2 [min: 1, max: 27]; average time points per patients for the adjusted model: 4.0 [min: 1, max: 27].*p-value < 0.05

### Achievement of glycemic target during the follow-up stratified by sex and age

**Fig. 3:**
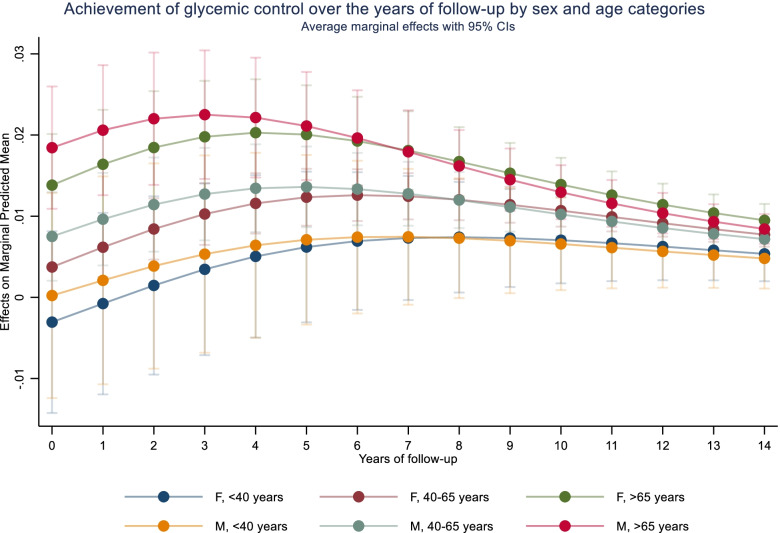
Probabilities and 95% confidence intervals for the achievement of glycemic control at specific time points by sex and age categories. This graph represents average marginal effects (the annual marginal predicted mean and 95% confidence interval) for achieving the glycemic target at specific time points over the years of the follow-up by sex and age category. In this representation, older individuals present a higher probability of achieving glycemic targets, particularly during the first years of the follow-up, while younger patients’ probability does not appear to change much with increasing time. Towards the end of follow-up, the probability was extremely low for all patients

The multi-level random-effects logistic regression stratified analyses based on sex and age categories for the achievement of glycemic target over the years of follow-up are presented in Table 3 and their average marginal effects in Table S3 (additional file). These findings revealed that only patients with an age > 40 years, independently of sex, increased their odds of achieving glycemic target over time. Per year of follow-up, for those between 40 and 65 years, it increased by approximately 12.0% (OR: 1.120, 95% CI: 1.070 to 1.173) for women and 13.5% (OR: 1.135, 95% CI: 1.072 to 1.200) for men, and for the age group of > 65 years, it increased by 14.1% (OR: 1.141, 95% CI: 1.050 to 1.240) for women and 21.6% (OR: 1.216, 95% CI: 1.104 to 1.340) for men. No change was observed among younger patients (< 40 years) associated with the increase of years of follow-up, but a fewer number of patients were observed within these age groups: 219 for men and 506 for women. In agreement with these findings, Fig. 3 exemplifies the average marginal effects and 95% CI for the achievement of glycemic control at specific time points over the follow-up by sex and age categories. Older individuals present a higher probability of achieving glycemic targets over the first years of follow-up while younger patients’ probability does not appear to change with increasing time. Nevertheless, towards the end of follow-up, the probability of achieving glycemic target was extremely low for all patients.

**Table 3:** Achievement of glycemic target by sex and age categories* (odds ratio and 95% confidence interval)*

	< 40 years		40–65 years		> 65 years	
	Women	Men	Women	Men	Women	Men
*N* patients (*N* observations)	506 (1812)	219 (764)	4197 (18,078)	1910 (7489)	1113 (4546)	698 (2799)
**Years follow-up**	1.123 (0.956, 1.318)	0.954 (0.740, 1.231)	1.120 (1.070, 1.173)	1.135 (1.072, 1.200)	1.141 (1.050, 1.240)	1.216 (1.104, 1.340)
	0.157	0.720	< 0.001*	< 0.001*	0.002*	< 0.001
**BMI**						
Overweight	0.964 (0.632, 1.469)	1.544 (0.919, 2.594)	1.315 (1.140, 1.517)	1.357 (1.137, 1.619)	0.950 (0.735, 1.190)	1.568 (1.162, 2.115)
	0.863	0.101	< 0.001*	0.001*	0.531	0.003*
Obese	1.911 (1.170, 3.123)	1.159 (0.486, 2.717)	1.225 (1.039, 1.444)	1.605 (1.208, 2.131)	1.028 (0.748, 1.412)	1.373 (0.788, 2.391)
	0.010	0.751	0.016	0.001*	0.864	0.263
**SBP**						
SBP < 130 mmHg	1.015 (0.713, 1.446)	0.609 (0.380, 0.974)	0.986 (0.879, 1.106)	0.878 (0.750, 1.028)	1.014 (0.802, 1.282)	0.910 (0.696, 1.191)
	0.932	0.039	0.815	0.105	0.907	0.494
**Initial treatment**						
OGLDs	1.812 (1.138, 2.884)	1.295 (0.688, 2.437)	2.579 (2.228, 2.985)	1.507 (1.241, 1.830)	1.846 (1.407, 2.423)	2.143 (1.540, 2.983)
	0.012	0.422	< 0.001*	< 0.001*	< 0.001*	< 0.001*
OGLDs + Insulin	0.875 (0.387, 1.981)	2.413 (0.989, 5.887)	1.267 (0.982, 1.635)	1.204 (0.841, 1.726)	0.610 (0.330, 1.126)	1.438 (0.759, 2.723)
	0.749	0.053	0.069	0.311	0.114	0.265
Diet	3.326 (0.979, 11.305)	-	11.267 (7.782, 16.312)	5.344 (3.425, 8.338)	6.096 (3.776, 9.840)	10.328 (5.234, 20.379)
	0.054		< 0.001*	< 0.001*	< 0.001*	< 0.001*
**Time-interactions**						
Overweight*years follow-up	1.032 (0.864, 1.232)	0.885 (0.710, 1.104)	0.969 (0.925, 1.015)	0.925 (0.869, 0.986)	1.028 (0.933, 1.132)	0.901 (0.801, 1.013)
	0.730	0.279	0.183	0.016	0.579	0.081
Obesity*years follow-up	0.809 (0.648, 1.009)	0.925 (0.572, 1.494)	0.961 (0.908, 1.017)	0.951 (0.860, 1.052)	0.987 (0.865, 1.126)	0.879 (0.724 1.068)
	0.060	0.749	0.172	0.328	0.846	0.195
SBP*years follow-up	0.949 (0.844, 1.069)	1.203 (0.976, 1.483)	1.021 (0.984, 1.060)	1.019 (0.966, 1.076)	0.991 (0.907, 1.083)	0.971 (0.881, 1.079)
	0.394	0.083	0.270	0.488	0.843	0.549
OGLDs*year follow-up	0.917 (0.767, 1.095)	1.049 (0.778, 1.413)	0.841 (0.798, 0.885)	0.891 (0.829, 0.959)	0.881 (0.788, 0.985)	0.873 (0.772, 0.989)
	0.337	0.755	< 0.001*	0.002*	0.026	0.032
OGLDs + Insulin*year follow-up	0.893 (0.635, 1.255)	0.448 (0.216, 0.927)	0.961 (0.865, 1.067)	1.051 (0.891, 1.240)	1.469 (1.079, 1.999)	0.887 (0.688, 1.143)
	0.514	0.031	0.451	0.552	0.015	0.354
Diet*year follow-up	0.545 (0.211, 1.409)	-	0.852 (0.732, 0.992)	0.917 (0.742, 1.133)	1.059 (0.816, 1.372)	0.934 (0.564, 1.547)
	0.210		0.039	0.424	0.665	0.792
*Variance intercept*^*+*^*(health centre)*	*-*	*-*	*0.059 (0.026, 1.132)*	*0.024 (0.007, 0.081)*	*0.029 (0.004, 0.195)*	*-*
*Variance intercept*^*+*^*(ID participant)*	*1.201 (0.779, 1.853)*	*0.427 (0.117, 1.556)*	*1.453 (1.274, 1.657)*	*0.859 (0.675, 1.092)*	*1.086 (0.802, 1.471)*	*1.197 (0.858, 1.671)*
*Variance slope*^*+*^*(years follow-up)*	*0.009 (0.000, 6.555)*	*0.079 (0.013, 0.483)*	*0.038 (0.024, 0.070)*	*0.029 (0.014, 0.059)*	*0.063 (0.033, 0.120)*	*0.043 (0.016, 0.111)*

*Abbreviations*: *BMI* body mass index, *OGLDs* oral glucose lowering drugs, *SBP* systolic blood pressure

Odds ratio (OR) and 95% confidence interval (95% CI) were obtained from a multi-level mixed-effects logistic regression with two random intercepts (health centres and participants ID) and one random slope (years of follow-up). The odds ratios’ interpretation is conditional on the random effects. Below every estimate with 95% CI, the corresponding *p*-value is presented

*Bonferroni-corrected *p*-value < 0.008. The model is adjusted only for baseline (first visit of follow-up) covariates (BMI, SBP, initial treatment and their corresponding interactions with years of follow-up). Achievement of glycemia target (achieved < 126 mg/dL and unachieved ≥ 126 mg/dL (reference)

^+^Reported as estimates and corresponding 95% CI. Reference categories for the covariates are the following: BMI (BMI < 25 kg/m2), SBP (elevated, > 130 mmHg), initial treatment (Insulin), and same reference group was used for the interaction of covariates with years of follow-up

## Discussion

This retrospective database study of routine data described demographic features and evaluated the achievement of glycemic control over the years of follow-up of more than 8000 adult patients with T2D attending health centres for disease follow-up in Kinshasa. In our study population, our findings revealed a higher proportion of women seeking treatment follow-up as well as a higher proportion of metabolic risk factors among women, particularly obesity and high blood pressure. We observed modest improvements in achieving the glycemic target over time.

Potential explanations for the demographic feature of the predominance of women (67%) in our study population comprise (1) women are more prone to seek health care and follow-up when ill, as supported by previous evidence in similar settings [23, 24], and (2) T2D affects more women than men in this context, potentially explained also by our finding of higher proportions of metabolic risk factors, particularly obesity, which is a well-known and a primary risk factor for the development of T2D [25]. In DRC or similar settings, the higher proportion of T2D in women remains controversial. A small cross-sectional study conducted in 2017 in Kinshasa aiming at quantifying the prevalence of determinants of psychological insulin resistance among patients with T2D attending often three diabetic centres observed that among 213 participants, 60.6% were female [26]; however, this was not confirmed by other studies conducted across different African settings. A systematic review and meta-analysis based on 36 cross-sectional studies conducted between 1983 and 2009 in SSA found a slightly higher prevalence of T2D in women (5.9%; 95% CI: 4.6–7.6) than in men (5.5%; 95% CI: 4.1–7.2) [27]. In contrast, another meta-analysis including 41 cross-sectional or population-based studies conducted between 2000 and 2015 across Africa found an overall prevalence of T2D of 13.7% in adults > 55 years old but the prevalence did not differ significantly by sex [28], as was also reported by the WHO in DRC’s country profile of 2016 (women 4.3% and men 4.2%) [29]. Our findings concerning the higher proportion of metabolic risk factors, overweight, obesity, and elevated SBP, among women are aligned with the existing body of evidence in the DRC and Africa [30–34]. In this regard, further research is needed to understand if the higher proportion of women attending health centres for T2D follow-up is related to seeking care more often or a matter of a higher prevalence of diabetes potentially linked to a higher prevalence of risk factors. Our findings also highlight the necessity for the implementation of health promotion strategies, particularly for women, targeting modifiable risk factors (e.g. optimal diet, promotion of physical activity, weight management) for cardiometabolic diseases.

We observed a modest improvement towards the achievement of the glycemic target over the years of follow-up. Particularly, higher probabilities of achieving glycemic control were seen over the first years of follow-up followed by a decline towards a longer follow-up. However, glycemic control remained poor overall. Poor glycemic control (HbA1c ≥ 7% and/or FPG ≥ 126 mg/dL) has been observed in reports of previous observational cross-sectional studies using hospital or primary care data from the DRC [10, 35], South Africa [12], and Ethiopia reporting a glycemic control achieved in at most one-fourth to one-third of the population [11]. A narrative study also suggested poor glycemic control across SSA countries due to poor knowledge of T2D in patients [36]. Evidence has also reported inadequate monitoring of disease-related complications regardless of chronic care programmes in place, hence stressing the importance of incorporating self-monitoring strategies to reduce T2D-related complications [37]. Despite differences in study methods (study design, parameters of comparison and sample size), there is a clear indication of poor glycemic control across primary care facilities in Africa. To advance from these findings, future research should align with The Lancet Commission on diabetes report describing key points to improve diabetes care in LMICs [38] as well as comprehensively evaluate the evolution and control of T2D through strong study designs with sufficiently long follow-up periods, using reliable and complementary indicators (e.g. HbA1c), monitor T2D-related comorbidities and promote self-management. This approach will allow for the translation of clear messages derived from research to patients, health providers, and policy-makers influencers.

The median duration of 1.8 years of follow-up within a time frame of almost 30 years suggests that one of the main aims of the project Kin-Antwerp, to have a better tool to monitor and improve retention in care of patients, has not been reached. Interviews with patients during field visits provided some service-related and person-related factors such as the long travel and waiting times for consultations, the concurrence with other duties in life, and lack of acquaintances to guide them to the clinic. Literature from other chronic care projects in similar settings points to migration linked to seasonal labour [39]) and financial and other priorities that interfere with self-management and empowerment [40]. As for a key element of a chronic care programme, it is essential is to retain retention patients in care in the control of their disease, further in-depth assessment of Kin-Antwerp is necessary to understand the motives why closer follow-up was not achieved, identify areas for improvement, and assess the continuation of the project.

Evidence has reported that the management of T2D in LMICs is often suboptimal due to challenges related to accessibility/availability of medication and has emphasised the need for T2D management guidelines to be tailored to constrained resources [13] and also to consider the patients’ proximal environment [41]. These barriers are also relevant in the DRC context suggesting that the limited accessibility and availability of care and treatments, particularly driven by financial barriers of the health system, may play key roles in poor glycemic control and follow-up.

Aiming at identifying subgroups of patients that may have difficulties in attaining glycemic targets and may require prioritised attention, stratified analyses revealed that those patients with an age <  40 years independently of their sex seem to have lower odds of achieving glycemic target over time. Overweight and obesity may also play a role in suboptimal glycemic control. This is in agreement with previous evidence in which younger age has been identified as a predictor of poor glycemic control in Ethiopia [11], Uganda [42], and other settings [43]. Also, literature has suggested a positive association between overweight/obesity and suboptimal glycemic control [44, 45]. We suggest further research on identifying the drivers of poor glycemic control in these subgroups to be able to develop tailored and effective strategies.

As our research entails a large study population, more than 8000 patients, and a 30-year follow-up time frame, it allowed us to provide an overall insight into the demographic characteristics and disease control of a study population participating in the Kin-Antwerp project. Another strength includes the advanced subgroup analyses conducted in this study that will potentially inform and improve strategies for the prevention and control of T2D. This study has also limitations. Findings are based on FPG and measured by a glucometer; hence, our results might not be completely representative and reliable of overall glycemic control of these patients and should be interpreted with caution; however, in the context of DRC, the recommended disease monitoring of high-income countries, including HbA1c, continuous glucose monitoring, and self-monitoring blood glucose at multiple instances a day, is not common nor affordable practice. Conclusions regarding the treatments could not be made, as this information was insufficient and any discussion would be too speculative. Our study sample population is not representative of the general patients with T2D in DRC; thus, our findings may not be generalisable to other populations. Also, it is important to acknowledge the measurements and registration errors by physicians/nurses in the written forms, as well as data-entry errors in the database. With the information available in the database, we cannot exclude the presence of residual confounding in our models as some important variables influencing glycemic control such as lifestyle behaviours (diet, exercise) or demographics such as economic status, were not registered in the database.

## Conclusion

This study sheds light on the disease control of more than 8000 patients with T2D followed within a time frame of almost 30 years in Kinshasa. Our findings highlight the poor glycemic control as well as the short duration of disease follow-up. Younger (< 40 years) patients seem to require prioritised attention to achieve the glycemic targets. Aligned with these findings, we recommend a deeper look into the Kin-Antwerp programme to understand what are the barriers to optimal effectiveness, identify key improvement areas, and explore the needs for continuation. Regardless, additional efforts for diabetes management guided by The Lancet Commission on diabetes report are necessary, particularly the introduction of reliable indicators (HbA1c) for disease monitoring along with self-management strategies including promotion of optimal lifestyle and treatment adherence.

## Supplementary Information

**Additional file 1: Image S1**. Follow-up paper-based form for diabetes in DRC. **Figure S1**. Histogram representing the distribution of the number of follow-up visits. **Figure S2**. Histogram representing the distribution of the years of follow-up. **Table S1**. Baseline characteristics stratified by sex and age group (subset of patients with a minimum of 1.5 years of follow-up). **Table S2**. Last follow-up characteristics stratified by sex and age group (subset of patients with a minimum of 1.5 years of follow-up). **Table S3**. Stratified analysis by sex and age categories for the achievement of glycemic target over the follow up time (Average marginal effects -estimated probabilities and 95% confidence interval derived from the multi-level random effect logistic model).

## Data Availability

Data used for this research is owned by Centre d’Éducation Diabète & Santé and currently is not publicly available. Data may be made available from the owner on reasonable request.

## Abbreviations

95% CI: 95% confidence interval
BMI: Body mass index
DBP: Diastolic blood pressure
DRC: The Democratic Republic of the Congo
FPG: Fasting plasma glucose
HbA1c: Haemoglobin A1c
IDF: International Diabetes Federation
IQR: Interquartile range
ITM: Institute of Tropical Medicine
LMICs: Low- and middle-income countries
OGLDs: Oral glucose lowering drugs
OR: Odds ratio
SBP: Systolic blood pressure
SD: Standard deviation
SSA: Sub-Saharan Africa
T2D: Type 2 diabetes
WHO: World Health Organisation
