# Supplementary material for: A retrospective database study of the demographic features and glycemic control of patients with type 2 diabetes in Kinshasa, Democratic Republic of the Congo
Source: BMC Med. 2022 Aug 19;20:258. doi: 10.1186/s12916-022-02458-2 (PMC9389797; doi:10.1186/s12916-022-02458-2)
Supplement: Supplementary file 1 — Additional file 1: Image S1. Follow-up paper-based form for diabetes in DRC. Figure S1. Histogram representing the distribution of the number of follow-up visits. Figure S2. Histogram representing the distribution of the years of follow-up. Table S1. Baseline characteristics stratified by sex and age group (subset of patients with a minimum of 1.5 years of follow-up). Table S2. Last follow-up characteristics stratified by sex and age group (subset of patients with a minimum of 1.5 years of follow-up). Table S3. Stratified analysis by sex and age categories for the achievement of glycemic target over the follow up time (Average marginal effects -estimated probabilities and 95% confidence interval derived from the multi-level random effect logistic model). [file 12916_2022_2458_MOESM1_ESM.pdf]

A retrospective database analysis of the demographic features and glycemic control of patients with type 2 diabetes in Kinshasa, Democratic Republic of the Congo.

Diana Sagastume[1*], Elly Mertens[1], Deogratias Katsuva Sibongwere[1], Jean-Claude Dimbelolo[2], Jean Clovis Kalobu Kabundi[3], Jeroen de Man[4], Josefien Van Olmen[4], José L. Peñalvo[1]

[1] Unit of Non-Communicable Diseases, Department of Public Health, Institute of Tropical Medicine, Nationalestraat 155 - 2000 Antwerp, Belgium

[2] Centre d'Éducation Diabète & Santé Boulevard Lumumba n° 1 Musoso district, Municipality of Limete, Kinshasa, République Démocratique du Congo

[3] Memisa, 19 Square de Meeûs, 1050 Brussels, Belgium and Memisa representation in Kinshasa, 47 Kisangani, commune de la Gombe, Kinshasa, République Démocratique du Congo

[4] Department of Family Medicine and Population Health, University of Antwerp, Doornstraat 331 - 2610 Wilrijk, Belgium

***Correspondence:**

Diana Sagastume, Institute of Tropical Medicine, Nationalestraat 155, 2000 Antwerp, Belgium

Email: dsagastume@itg.be

Table of content

Image S1. Follow-up paper-based form for diabetes in DRC 3

Figure S1. Histogram representing the distribution of the number of follow-up visits. 4

Figure S2. Histogram representing the distribution of the years of follow-up. 4

Table S1. Baseline characteristics stratified by sex and age group (subset of patients with a minimum of 1.5 years of follow-up) 5

Table S2. Last follow-up characteristics stratified by sex and age group (subset of patients with a minimum of 1.5 years of follow-up) 6

Table S3. Stratified analysis by sex and age categories for the achievement of glycemic target over the follow up time (Average marginal effects -estimated probabilities and 95% confidence interval derived from the multi-level random effect logistic model) 7

Image S1. Follow-up paper-based form for diabetes in DRC

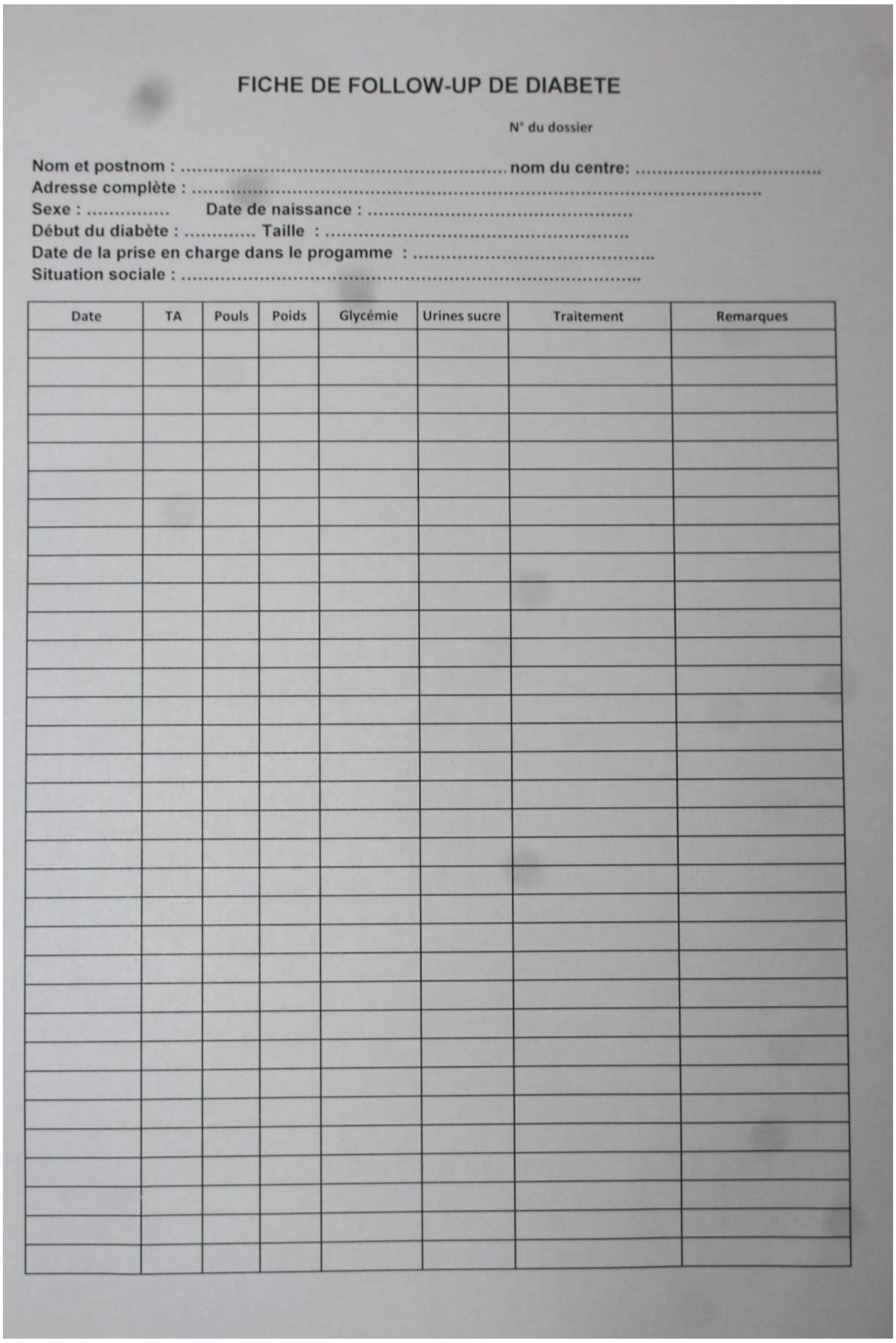

FICHE DE FOLLOW-UP DE DIABETE

N° du dossier

Nom et postnom : .... nom du centre: ....
Adresse complète : ....
Sexe : .... Date de naissance : ....
Début du diabète : .... Taille : ....
Date de la prise en charge dans le progamme : ....
Situation sociale : ....

Date	TA	Pouls	Poids	Glycémie	Urines sucre	Traitement	Remarques

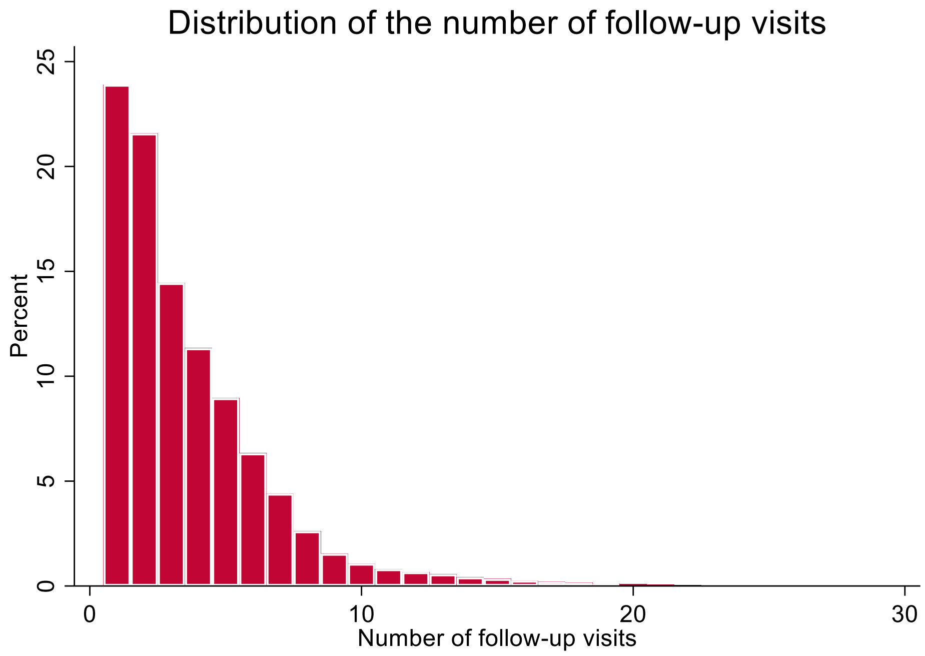

Figure S1. Histogram representing the distribution of the number of follow-up visits.

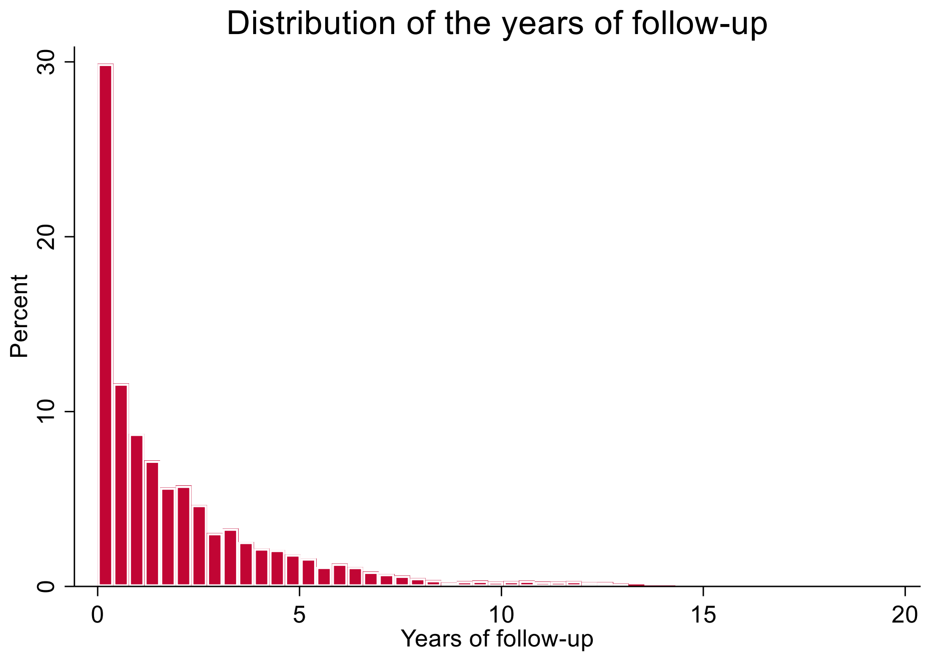

Figure S2. Histogram representing the distribution of the years of follow-up.

Table S1. Baseline characteristics stratified by sex and age group (subset of patients with a minimum of 1.5 years of follow-up)

		<40 years		40-65 years		>65 years	
		**Women**	**Men**	**Women**	**Men**	**Women**	**Men**
N patients		256	104	2,531	1,032	616	396
**Initial treatment** *n(%)*							
	OGLDs	63 (24.6)	24 (23.1)	924 (36.5)	322 (31.2)	213 (34.6)	132 (33.3)
	Insulin	171 (66.8)	73 (70.2)	1,366 (54.0)	621 (60.2)	346 (56.2)	227 (57.3)
	Diet	4 (1.6)	1 (1.0)	56 (2.2)	20 (1.9)	24 (3.9)	14 (3.5)
	OGLDs + Insulin	18 (7.0)	6 (5.7)	185 (7.3)	69 (6.7)	33 (5.3)	23 (5.8)
**Anthropometry**							
**Weight** *mean ± SD*	*Kg*	67.6 ± 14.0	68.7 ± 15.0	70.2 ± 14.0	70.7 ± 13.5	65.0 ± 13.1	66.4 ± 12.9
**BMI** *mean ± SD*	*kg/m^2*	25.4 ± 5.2	24.0 ± 2.8	27.0 ± 5.4	24.3 ± 4.1	25.9 ± 5.1	23.6 ± 3.9
**BMI classification** *n (%)*	*Underweight/normal (<24.9)*	106 (42.6)	56 (54.4)	808 (32.6)	529 (52.9)	238 (39.7)	230 (60.2)
	Overweight (25-29.9)	78 (31.3)	34 (33.0)	975 (39.3)	382 (38.2)	221 (36.8)	127 (33.3)
	Obese (≥ 30)	65 (26.1)	13 (12.6)	698 (28.1)	89 (8.9)	141 (23.5)	25 (6.5)
**Clinical parameters**							
**SBP** *mean ± SD*	*mmHg*	120.5 ± 23.0	119.0 ± 19.2	131.1 ± 23.7	128.2 ± 22.0	140.4 ± 25.9	132 ± 22.0
**SBP classification** *n (%)*	*Normal (<130 mmHg)*	175 (69.2)	70 (70)	1,139 (45.6)	507 (49.9)	199 (32.7)	162 (41.4)
	Elevated (≥130 <140 mmHg)	26 (10.3)	12 (12)	379 (15.2)	149 (14.7)	81 (13.3)	50 (12.8)
	High (≥140 mmHg)	52 (20.5)	18 (18)	980 (39.2)	360 (35.4)	328 (54.0)	179 (45.8)
**FPG** *median[IQR]*	*mg/dL*	227 [147 – 308.5]	206 [135.5 – 339.5]	216 [144 – 315]	215.5 [142 – 320]	196 [137 – 296.5}	193 [126.5 - 305]
**Glycemic target** *n (%)*	*Achieved (FPG <126 mg/dL)*	211 (82.4)	86 (82.7)	2,116 (83.6)	856 (83.0)	498 (80.8)	301 (76.0)
	Unachieved FPG (≥126 mg/dL)	45 (17.6)	18 (17.3)	415 (16.4)	176 (17.0)	118 (19.2)	95 (24.0)

Abbreviations: Body mass index (BMI); Fasting plasma glucose (FPG); Oral glucose lowering drugs (OGLDs); Systolic blood pressure (SBP). For those <40 years old and women the denominator for initial treatment (256), weight (253), BMI and BMI classification (249), SBP and SBP classification (253), FPG and glycemic target (256); <40 years old and men the denominator for initial treatment (104), weight (104), BMI and BMI classification (103), SBP and SBP classification (100), FPG and glycemic target (104); 40-65 years old and women the denominator for initial treatment (2,531), weight (2,508), BMI and BMI classification (2,444), SBP and SBP classification (2,498), FPG and glycemic target (2,531); 40-65 years old and men the denominator for initial treatment (1,032), weight (1011), BMI and BMI classification (1,000), SBP and SBP classification (1,016), FPG and glycemic target (1,032); >65 years old and women the denominator for initial treatment (616), weight (610), BMI and BMI classification (589), SBP and SBP classification (608), FPG and glycemic target (616); >65 years old and men the denominator for initial treatment (396), weight (386), BMI and BMI classification (382), SBP and SBP classification (391), FPG and glycemic target (396).

Table S2. Last follow-up characteristics stratified by sex and age group (subset of patients with a minimum of 1.5 years of follow-up)

		**<40 years**		**40-65 years**		**>65 years**	
		**Women**	**Men**	**Women**	**Men**	**Women**	**Men**
N patients		256	104	2,531	1,032	616	396
**Treatment last follow-up visit** *n(%)*							
	OGLDs	57 (22.3)	22 (21.6)	1,000 (40.0)	369 (36.2)	248 (40.6)	164 (42.4)
	Insulin	154 (60.4)	65 (63.7)	1,036 (41.5)	496 (48.6)	263 (43.1)	173 (44.7)
	Diet	12 (4.7)	2 (2.0)	103 (4.1)	36 (3.5)	46 (7.5)	28 (7.2)
	OGLDs + Insulin	32 (12.6)	13 (12.7)	361 (14.4)	119 (11.7)	54 (8.8)	22 (5.7)
**Anthropometry**							
**Weight** *mean ± SD*	*Kg*	68.1 ± 12.8	69.8 ± 16.4	69.9 ± 13.8	71.2 ± 13.1	64.2 ± 13.0	65.2 ± 12.3
**BMI** *mean ± SD*	*kg/m²*	26.4 ± 4.7	24.7 ± 4.7	27.4 ± 5.1	25.0 ± 4.1	26.1 ± 4.9	23.8 ± 3.9
**BMI classification** *n (%)*	*Underweight/normal (<24.9)*	98 (40.3)	57 (56.5)	816 (34.0)	513 (52.0)	255 (44.2)	238 (63.5)
	Overweight (25-29.9)	87 (35.8)	28 (27.7)	915 (38.2	359 (36.4)	206 (35.7)	118 (31.4)
	Obese (≥ 30)	58 (23.9)	16 (15.8)	665 (27.8)	115 (11.6)	116 (20.1)	19 (5.1)
**Clinical parameters**							
**SBP** *mean ± SD*	*mmHg*	125.0 ± 22.2	122.7 ± 17.9	135.5 ± 23.6	134.3 ± 22.8	142.7 ± 26.6	136.6 ± 22.7
**SBP classification** *n (%)*	*Normal (<130 mmHg)*	148 (59.0)	58 (57.4)	973 (38.9)	391 (38.1)	169 (27.7)	130 (33.3)
	Elevated (≥130 <140 mmHg)	33 (13.1)	21 (20.8)	368 (14.7)	173 (16.9)	82 (13.4)	63 (16.1)
	High (≥140 mmHg)	70 (27.9)	22 (21.8)	1,160 (46.4)	461 (45.0)	359 (58.9)	198 (50.6)
**FPG** *median[IQR]*	*mg/dL*	173.5 [120.5 – 249.5]	175 [132 – 241]	161.5 [122 – 229]	254 [115 – 217]	152 [115 – 204}	140.5 [109 - 195]
**Glycemic target** *n (%)*	*Achieved (FPG <126 mg/dL)*	182 (72.2)	82 (78.9)	1,809 (72.3)	685 (67.0)	403 (66.1)	241 (61.5)
	Unachieved FPG (≥126 mg/dL)	70 (27.8)	22 (21.1)	699 (27.9)	337 (33.0)	207 (33.9)	151 (38.5)

Abbreviations: Body mass index (BMI); Fasting plasma glucose (FPG); Oral glucose lowering drugs (OGLDs); Systolic blood pressure (SBP). For those <40 years old and women the denominator for initial treatment (255), weight (247), BMI and BMI classification (243), SBP and SBP classification (251), FPG and glycemic target (252); <40 years old and men the denominator for initial treatment (102), weight (102), BMI and BMI classification (101), SBP and SBP classification (101), FPG and glycemic target (104); 40-65 years old and women the denominator for initial treatment (2,500), weight (2,424), BMI and BMI classification (2,396), SBP and SBP classification (2,501), FPG and glycemic target (2,508); 40-65 years old and men the denominator for initial treatment (1,020), weight (999), BMI and BMI classification (987), SBP and SBP classification (1,025), FPG and glycemic target (1,022); >65 years old and women the denominator for initial treatment (611), weight (587), BMI and BMI classification (577), SBP and SBP classification (610), FPG and glycemic target (610); >65 years old and men the denominator for initial treatment (387), weight (381), BMI and BMI classification (375), SBP and SBP classification (391), FPG and glycemic target (392).

Table S3. Stratified analysis by sex and age categories for the achievement of glycemic target over the follow up time (Average marginal effects -estimated probabilities and 95% confidence interval derived from the multi-level random effect logistic model)

	<40 years		40-65 years		>65 years	
	**Women**	**Men**	**Women**	**Men**	**Women**	**Men**
N patients (N observations)	506 (1,812)	219 (764)	4,197 (18,078)	1,910 (7,489)	1,113 (4,546)	698 (2,799)
**Years follow-up**	0.001 (-0.012, 0.014)<br>0.992	-0.002 (-0.026, 0.022)<br>0.878	0.006 (0.002, 0.009)<br>0.002	0.013 (0.007, 0.019)<br><0.001*	0.022 (0.014, 0.030)<br><0.001*	0.016 (0.006, 0.027)<br>0.003*
**BMI**						
Overweight	0.003 (-0.052, 0.058)<br>0.910	0.046 (-0.035, 0.126)<br>0.266	0.032 (0.014, 0.049)<br><0.001	0.027 (-0.001, 0.054)<br>0.040	0.000 (-0.034, 0.034)<br>0.992	0.046 (-0.001, 0.091)<br>0.046
Obese	0.047 (-0.020, 0.114)<br>0.166	-0.004 (-0.122, 0.129)<br>0.955	0.018 (-0.003, 0.039)<br>0.092	0.068 (0.025, 0.111)<br>0.002*	0.000 (-0.044, 0.045)<br>0.982	0.014 (-0.068, 0.095)<br>0.744
**SBP**						
	-0.012 (-0.056, 0.032)<br>0.596	-0.040 (-0.111, 0.032)<br>0.275	0.004 (-0.009, 0.018)<br>0.529	-0.016 (-0.039, 0.006)<br>0.153	-0.000 (-0.031, -0.030)<br>0.973	-0.027 (-0.065, 0.011)<br>0.168
**Initial treatment**						
OGLDs	0.072 (0.004, 0.140)<br>0.038	0.061 (-0.042, 0.167)<br>0.254	0.092 (0.072, 0.113)<br><0.001*	0.034 (0.003, 0.065)<br>0.032	0.063 (0.021, 0.104)<br>0.003	0.093 (0.036, 0.150)<br>0.001*
OGLDs + Insulin	-0.047 (-0.143, 0.049)<br>0.334	-0.009 (-0.136, 0.118)<br>0.890	0.022 (-0.014, 0.058)<br>0.234	0.051 (-0.008, 0.109)<br>0.088	0.043 (-0.040, 0.127)<br>0.311	0.024 (-0.085, 0.134)<br>0.660
Diet	0.059 (-0.125, 0.243)<br>0.529	-	0.362 (0.297, 0.428)<br><0.001*	0.294 (0.202, 0.386)<br><0.001*	0.346 (0.256, 0.435)<br><0.001*	0.396 (0.28, 0.525)<br><0.001*

Abbreviations: Body mass index (BMI); Oral glucose lowering drugs (OGLDs); Estimated probabilities and 95% confidence intervals of the predicted marginal effects from the multi-level mixed-effects logistic. Achievement of glycemia target (achieved < 126 mg/dL and unachieved ≥ 126 mg/dL (reference). Below every probabilities and 95%CI, the corresponding p-value is presented. **Bonferroni-corrected p-value <0.008.* The model is adjusted only for baseline (first visit of follow-up) covariates (BMI, SBP, initial treatment). Reference categories for the covariates are the following: BMI (BMI <25 kg/m^2), SBP (elevated, >130 mmHg), initial treatment (Insulin).
